# An audit during COVID-19: monitoring of CMHT-patient contact and physical health assessments in a rural Welsh setting

**DOI:** 10.1192/bjo.2021.263

**Published:** 2021-06-18

**Authors:** Shreya Jauhari, Fran Foster

**Affiliations:** 1 King's College London; 2 Powys Teaching Health Board

## Abstract

**Aims:**

The enforcement of lockdowns and restrictions on non-essential contact have changed Community Mental Health Team (CMHT) practice. Therefore, this audit carried out its 4th cycle of physical health monitoring for patients on antipsychotics with severe mental illness (SMI) under the CMHT during the period of the COVID-19 pandemic in order to observe its impact on physical health monitoring. In addition, with the increased use of telepsychiatry substituting routine face-to-face appointments during the pandemic, this audit also reviews the effect of lockdown on maintenance of contact between CMHT and people with SMI.

Primary Objective: to compare the current clinical practice with the standards derived from NICE guidelines which include parameters like weight, body-mass index, blood pressure, ECG and blood tests, then compare with the previous three audit cycles, which collected identical data.

Secondary Objective: to monitor amount of contact between healthcare staff and people with SMI on antipsychotics during the three months of Welsh lockdown and compare current clinical practice with the clinical practice achieved in the identical period in 2019.

**Method:**

Method for Primary Objective: Clinical practice on physical health checks were split into 10 standards derived from the NICE guidelines (NICEQS80, Quality Standard 6). Data collection surrounding physical health checks of patients on antipsychotics from 26th June 2019 to 26th June 2020 were collected and compared with the previous three audit cycles, which collected identical data.

Method for Secondary Objective: Retrospective data surrounding amount and type of contact between CMHT and people with SMI was collected from 26th March 2020 to 26th June 2020, a period of enforced lockdown in Wales, and compared with the identical period in 2019.

**Result:**

The audit iterates trends over the last 4 cycles (2016/2017, 2017/2018, 2018/2019 and 2019/2020). The current audit cycle increased in 2/10 standards and decreased in 8/10 standards, compared with the average compliance in the 3 previous audit cycles. Out of the 10 derived standards, certain standards fared worse than others.

There was a 79% increase in the number of staff-patient contact during the lockdown period. The majority of the contact in 2019 was face-to-face (84.31%), however, as expected, in 2020 the majority of the contact was non face-to-face (61.75%). However, this was accompanied by an 85.79%

**Conclusion:**

Despite being in a pandemic, patient contact was maintained. Physical health monitoring has decreased in the majority of standards, therefore greater attention is needed to address this. Recommendations are provided in the audit.

